# Metastatic mucinous adenocarcinoma from breast mimicking a pyogenic granuloma in the gingiva: a case report

**DOI:** 10.11604/pamj.2024.47.14.36983

**Published:** 2024-01-11

**Authors:** Rudra Prasad Chatterjee, Sangeeta Sinha, Debdita Banerjee, Lamia Parveen, Sanchita Kundu

**Affiliations:** 1 Faculty of Oral and Maxillofacial Pathology, Department of Oral and Maxillofacial Pathology, Guru Nanak Institute of Dental Sciences and Research, Kolkata, India,; 2 Head and Neck Oncopathology, Narayana Superspeciality Hospital, Howrah, India,; 3 Burdwan Medical College, Bardhaman, India,; 4 The Mediview Clinic, Howrah, India

**Keywords:** Adenocarcinoma, mucinous, metastatic, pyogenic granuloma, case report

## Abstract

Mucin-producing adenocarcinomas (MAC) are an extremely rare, indistinct group of neoplasm having either a salivary gland origin or with prominent glandular component. The diagnosis is chiefly based on the histological aspect conjoined with immunohistochemical evaluation as clinico-radiographical features are non-specific. It can arise as a primary metastasis to soft tissues, most commonly from either lung, breast, kidney, or colon. This paper reports a 51-year-old woman with buccolingual gingival swelling having a final diagnosis of metastatic mucinous adenocarcinoma from the breast. A tissue biopsy was performed followed by immunohistochemistry that confirmed the diagnosis. They are extremely rare, making the diagnosis challenging as it may mimic a benign neoplasm. It accounts for approximately 1% of all oral malignant neoplasms having gingival propensity. The clinician should therefore take into account every diagnostic aspect while encountering such oral lesions to achieve proper patient welfare.

## Introduction

Salivary gland tumors are rare, complex heterogenous histopathological entities involving both major and minor salivary glands. These lesions, having both benign and malignant counterparts present with a wide range of varying etiopathogenesis, histopathology, and prognosis making diagnosis and management challenging. They account for 0.5 to 1.2% of all malignancies constituting 5% of head and neck. The World Health Organization (WHO) narrowed down to 20 entities in 2017 from 24 different entities [1]. The most common benign neoplasm is pleomorphic adenoma while the malignant series includes mucoepidermoid carcinoma, acinic cell carcinoma, adenoid cystic carcinoma, carcinoma ex-pleomorphic adenoma, and adenocarcinoma [2].

Salivary gland adenocarcinoma frequently shows prominent mucinous differentiation but not like mucoepidermoid carcinoma or the mucin-rich variant of salivary duct carcinoma making them exclusive and incoherent [3]. Mucinous adenocarcinoma is a rare malignant tumour composed of epithelial clusters within large pools of extracellular mucin that mostly occupy the lesion [4]. As mucinous differentiation was a nonspecific feature, in 2017 WHO grouped mucinous adenocarcinoma along with many entirely dissimilar tumors, into the broad category of adenocarcinoma not otherwise specified (NOS) [1,3]. This report presents a case of a 51-year-old woman with mucinous adenocarcinoma mimicking benign lesion along with its various clinical, histopathological, and diagnostic aspects.

## Patient and observation

**Patient information:** a 51-year-old woman, G1P1A0, non-smoker, non-alcoholic, non-hypertensive with a gingival swelling in the anterior region of the lower jaw.

**Figure 1:**
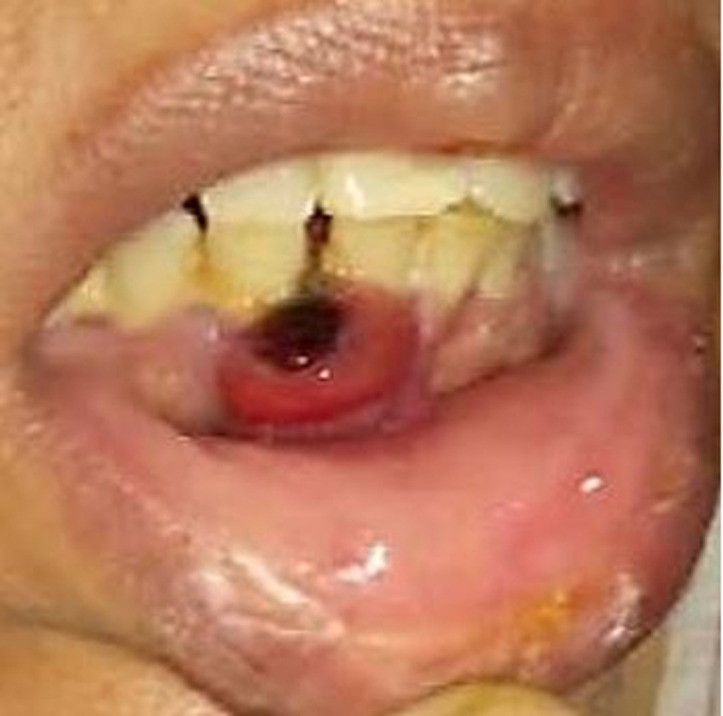
intra-oral photographs: presence of a medium-sized, round to irregular, bucco-lingual gingival swelling measuring about 2.5 x 1.5 cm having an erythematous overlying surface with a central dark area, extending from the distal aspect of the permanent right mandibular central to lateral incisor

**Clinical findings:** the patient presented with a medium-sized, soft to firm, round to irregular gingival (bucco-lingual) swelling having an erythematous surface with a central dark area, measuring about 2.5 x 1.5 cm, involving the mandibular right central and lateral incisor with concomitant mobility of the regional tooth (Figure 1). Extra-orally, the lower lip was everted due to the presence of the lesion.

**Timeline of the current episode:** on 21 April 2021: biopsy, histopathology. On 29 April 2021: a clinical review and additional general health history were taken (the patient gave a history of breast lesions which she thought of no importance earlier). On 13 May 2021: immuno-histochemical studies were conducted. On 19 May 2021: the patient referral to a medical oncologist.

**Figure 2:**
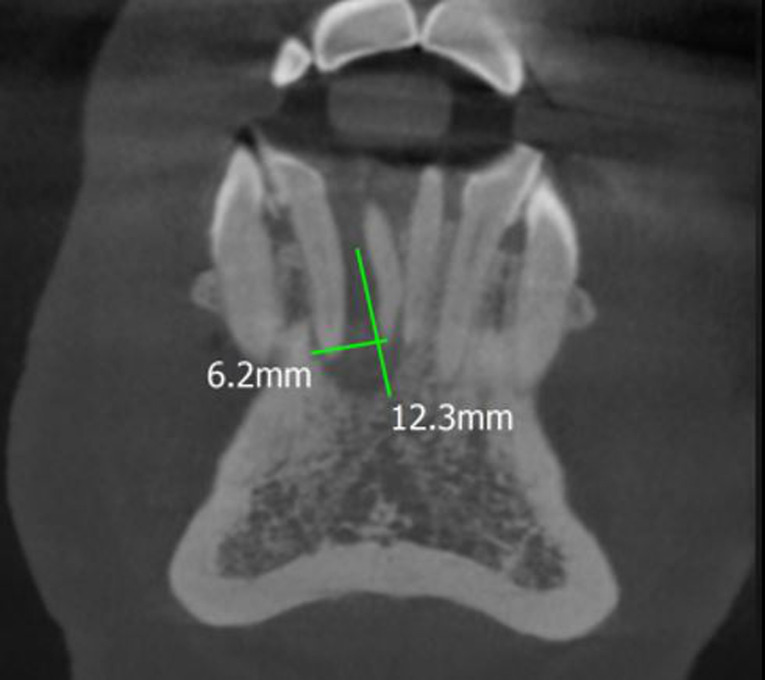
cone-beam computed tomography scan: peri-apical pathosis measuring about 12.3 x 6.2 mm with evidence of a bucco-lingual intra-bony osteolytic ill-defined area in relation to 41 and 42; presence of thin labial cortical plate was also noted

**Figure 3:**
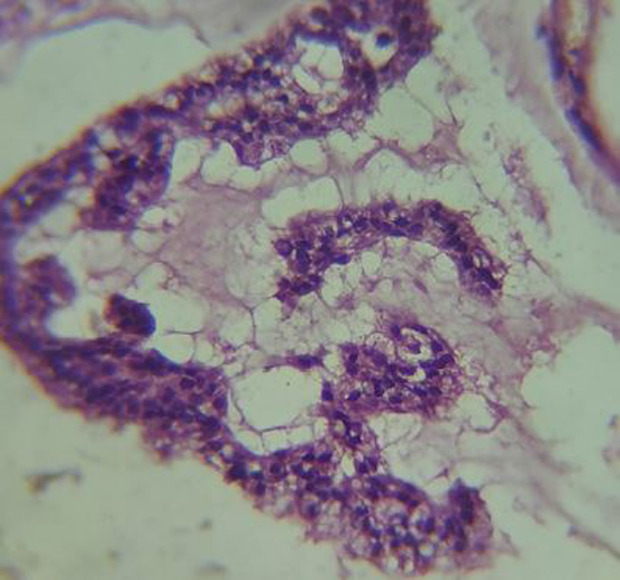
histopathological photomicrograph: multiple, glandular epithelial rests arranged in irregular strands having hyperchromatic nucleus and pleomorphism with tumour cell clusters floating in mucous-filled cystic cavities separated by connective fibrous strands [hematoxylin-Eosin staining x400 magnification]

**Figure 4:**
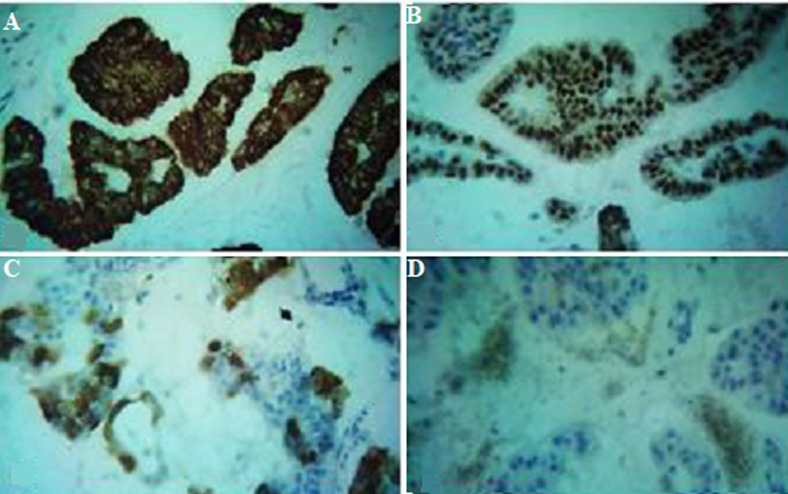
(A) positive immunohistochemistry (IHC) photomicrograph for CK 7; (B) positive for ER; (C) positive for mammoglobin; (D) focally positive for CEA (all slides at x400 magnification)

**Diagnostic assessment:** cone-beam computed tomography revealed: peri-apical pathosis measuring about 12.3 x 6.2 mm with a bucco-lingual intra-bony osteolytic ill-defined area in relation to 41 and 42 along with thinning of labial cortical plate (Figure 2). Biopsy and histopathology sections revealed multiple, glandular epithelial rests arranged in irregular strands having a hyperchromatic nucleus and pleomorphism with tumour cell clusters floating in mucous-filled cystic cavities separated by connective fibrous strands (Figure 3) suggesting: malignant salivary gland neoplasm favouring Adenocarcinoma. In between, the patient revealed about a solid lump-like mass on the lateral aspect of the left breast that was accidentally discovered on self-examination which she thought was of no importance earlier. Immunohistochemistry showed cytokeratin 7 (CK7) positive (Figure 4A), cytokeratin 20 (CK20) negative, estrogen receptor (ER) positive (Figure 4B), mammoglobin positive (Figure 4C), positive (focal) for gross cystic disease fluid protein 15 and carcinoembryonic antigen (Figure 4D).

**Diagnosis:** the results were consistent with breast cancer metastasis. A diagnosis of metastatic mucinous carcinoma, consistent with metastatic mucinous carcinoma from the breast was established.

**Therapeutic interventions:** the patient was called for necessary intervention and was immediately advised for consultation of an oncologist along with a positron emission tomography (PET) scan to evaluate organs and tissues for the presence of disease or any other related conditions.

**Follow-up and outcome of interventions:** due to the extreme severity of the diagnosis, the patient went to a higher and esteemed medical institute for necessary interventions. However, the patient did not revert back for a follow-up.

**Patient perspective:** *“My diagnosis is beyond my imagination and I intend to get cured completely and thereby I need to go a miles away for my survival”*.

**Informed consent:** written informed consent was obtained from the patient.

## Discussion

Mucin-producing adenocarcinomas (MAC) are an extremely rare, indistinct group of neoplasms [3]. They are commonly encountered in the colon, pancreas, ovary, lung, prostate, and breast. Unusual sites are the nasal cavity, paranasal sinuses, and mandibular ramus whereas salivary gland mucinous adenocarcinomas are extremely uncommon entities [5]. Metastatic MAC is also common with gingiva with mandible being the common site for jawbone metastases [6]. It accounts for about 1% of all oral malignant neoplasms. They resemble reactive lesions like pyogenic granuloma, peripheral giant cell granuloma, and fibrous epulis, especially in the early stages. Although some authors believe that fast expansile growth associated with gingival metastases is a distinctive feature which is also shown by pyogenic granulomas. Furthermore, oral metastases were discovered to be the first evidence of an unknown primary tumour in 25 to 37% of patients, making diagnosis even more challenging [7].

Men are more likely to be affected than women (ratio of 3: 1) with peak incidence between the fifth to sixth decade of life [4,5,8]. In this present case, the affected individual was a female with having age of 51 years but although her age group was consistent with other case reports, her gender was unusual. The affected individual usually presents with a painless exophytic growth, with the most common site affected being the gingiva [6,8]. In this case report also, the lesion was present in the anterior gingival region. Tumours display glandular proliferations with or without cyst formation. The morphology of the cells can either be cuboidal, columnar, polygonal, clear, mucinous, oncocytoid and plasmacytoid, exhibiting different patterns including small confluent nests or cords or large islands with intervening connective tissue and densely cellular stroma. It can be graded as low, intermediate, or high depending upon the degree of cellular atypia [1,6].

Histopathological evaluation revealed the presence of cells floating in mucin pooled areas, being separated by fibrous tissue and following the glandular irregular arrangement with cellular atypia, which helped us to diagnose the case as a malignant neoplasm. The mucinous areas stain positive with periodic acid-schiff, mucicarmine, and alcian blue [8]. Immunohistochemistry plays an important role in confirming the diagnosis. CK7 positive with negative CK 20 confirmed the diagnosis of mucinous adenocarcinoma. Various studies reported adenocarcinoma to be positive for CK7 (100%) and uniformly negative for CK20 [3]. CK 7(+)/CK 20(-) phenotype is considered as salivary diagnostic marker [5]. The reactivity for estrogen receptors strongly suggested breast primary whereas CEA positivity may vary [8]. It is one of the most aggressive types of malignancy having risks for early lymph node metastases, suggesting a possible role for elective neck management. It contrasts greatly with its cutaneous and mammary analogues which share an overall favourable prognosis [8].

The present case was initially diagnosed as a case of adenocarcinoma having a glandular component based on its histopathological features but the history of a solid lump in the breast of the patient led to diversification of our diagnostic gateway leading to the fact of a possible metastatic lesion. After immunohistochemical analysis, it was finally diagnosed as metastatic mucinous carcinoma, consistent with metastatic mucinous carcinoma from the breast.

## Conclusion

Mucinous adenocarcinoma is an elusive and controversial entity, encountered seldomly having diagnostic complexity. The term has been applied to the broader and heterogeneous group of “mucin-producing adenocarcinoma”, making it impossible to distinguish a primary and a metastatic MAC on histologic grounds alone. The mucinous appearance itself is not pathognomonic of MAC and many salivary gland carcinomas may exhibit this trait. Therefore, comprehensive clinical, physical, and radiological including computerized tomography scan and histopathological examinations needs to be performed along with immunohistochemistry being used as an adjunct for the final diagnosis.

## References

[1] El-Naggar AK, Chan JKC, Grandis JR, Takata T, Slootweg PJ (2017). WHO Classification of Head and Neck Tumours. World Health Organization.

[2] Young A, Okuyemi OT (2023). Malignant Salivary Gland Tumors. StatPearls [Internet].

[3] Rooper LM, Argyris PP, Thompson LD, Gagan J, Westra WH, Jordan RC (2021). Salivary Mucinous Adenocarcinoma Is a Histologically Diverse Single Entity With Recurrent AKT1 E17K Mutations: Clinicopathologic and Molecular Characterization With Proposal for a Unified Classification. Am J Surg Pathol.

[4] Barnes L, Eveson J, Reichart P, Sidransky D (2005). World Health Organization classification of tumours: pathology and genetics of head and neck tumours.

[5] Bhat SP, Prasad KH, Bhat VS, Aroor R (2014). Primary mucinous carcinoma of buccal mucosa: a rare case report. Indian J Surg Oncol.

[6] Nuyen BA, Tang CG (2016). Gingival Metastasis: A Case Report and Literature Review. Perm J.

[7] Seoane J, Varela-Centelles P, López-Niño J, Vázquez I, Abdulkader I, García-Caballero T (2010). Gingival mucinous adenocarcinoma of a minor salivary gland. J Periodontol.

[8] Ide F, Mishima K, Tanaka A, Saito I, Kusama K (2009). Mucinous adenocarcinoma of minor salivary glands: a high-grade malignancy prone to lymph node metastasis. Virchows Arch.

